# Spectroscopic tools for an automated suspension cell culture screening system

**DOI:** 10.1186/1753-6561-9-S9-P31

**Published:** 2015-12-14

**Authors:** Carsten Musmann, Klaus Joeris, Sven Markert

**Affiliations:** 1 Roche Diagnostics GmbH, Pharma Biotech Production and Development, Penzberg, Germany

## Background

We developed an automated, multiwell plate (MWP) based screening system for suspension cell cultures which is now routinely used in cell culture process development. It is characterized by a fully automated workflow with integrated analytical instrumentation. It uses shaken 6-24 well plates as bioreactors which can be run in batch and fed-batch mode with a capacity of up to 768 reactors in parallel [1,2]. A wide ranging analytical portfolio is available to monitor cell culture processes and also a cooperation with internal high throughput (HT) analytic groups to characterize product quality. The current work focuses on expanding the analytical portfolio to develop control mechanisms for automated cell culture processes. Besides setting up a robust method for pH measurement we evaluated different spectroscopic techniques like Raman or infrared as fast and powerful analytical tools.

## Results

Infrared and Raman spectroscopy are powerful tools for the simultaneous detection and quantification of several key components in cell culture processes. Non-invasive and near real-time measurement make them to ideal tools for small scale HT systems. By using Infrared spectroscopy some challenges must be overcome. Water, for example, has a high absorption in the infrared range and can mask the signals of important components. Generally, the low concentration of the target components presents in combination with the complex cell culture media a problem in the quantification process. Compared to the Raman spectroscopy the Infrared spectroscopy is more cost efficient and has higher signal intensities.

Also challenges have to be managed by using the Raman spectroscopy. As mentioned before for the Infrared spectroscopy, the low concentrations of the target components are in combination with the complex cell culture media a problem for the Raman spectroscopy, too. Additionally the low signal intensities and the high laser power, which is needed, might be a challenge. In comparison to the Infrared spectroscopy the Raman spectroscopy is useable for liquid samples without a strong signal of the water and fully automated Raman devices are available.

Despite these challenges we could show the quantification of different metabolites and nutrients of the cell culture process using these techniques. With the Infrared spectroscopy we were able to quantify the glucose, lactate, ammonia and antibody concentration. The application of the Raman spectroscopy could be shown for the glucose, lactate and antibody concentration determination.

**Figure 1:**
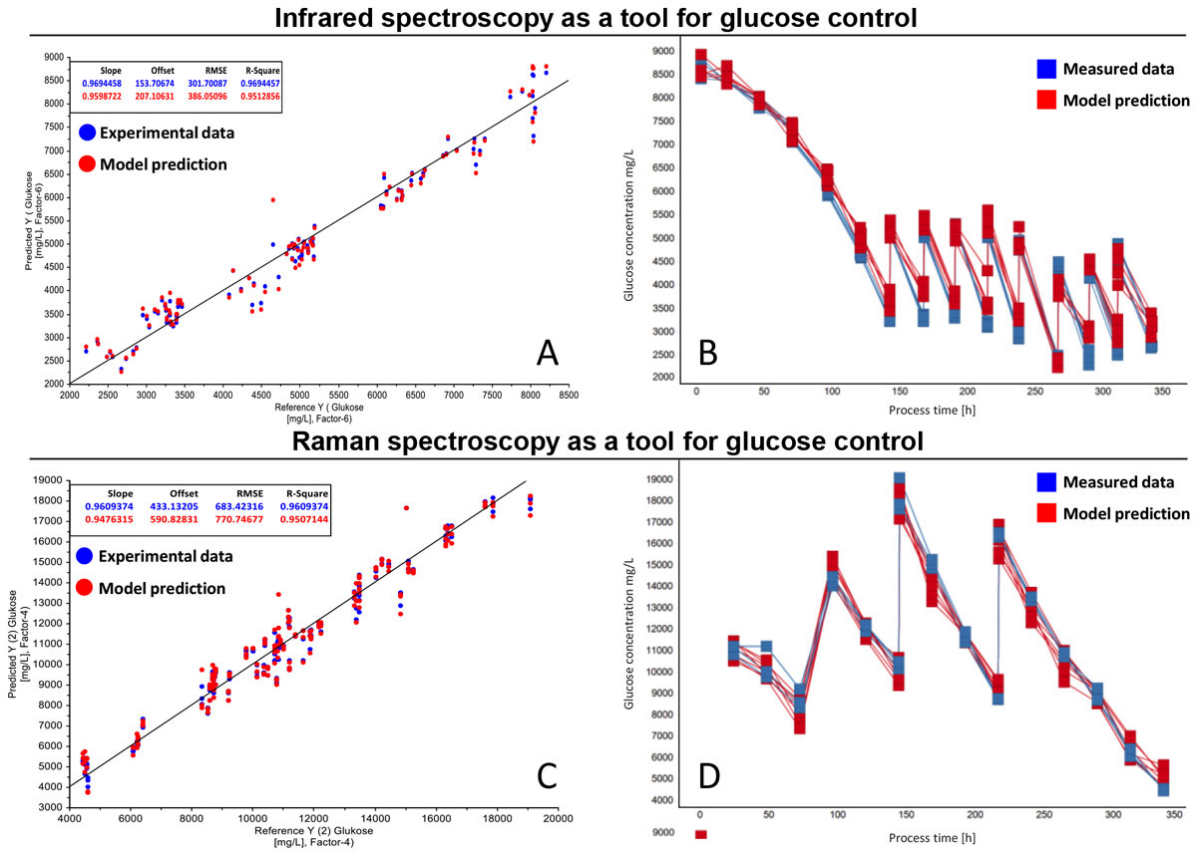
**A and C: Chemometric calibration model for glucose concentration using Infrared spectroscopy (a) and Raman spectroscopy (c)**. The model provides high precision and low deviations. The blue points represent the experimental data and the red points represent the respective model prediction. B and D: Application of the Infrared calibration model (b) and Raman calibration model (d) for monitoring and control of the glucose concentration in a cell culture process. The blue curves represent the measured data and the red curves the concentrations of the model prediction.

An important application of these spectroscopic methods for our automated suspension cell culture screening system is the control of the glucose concentration. We could show this application in fed-batch processes for different cell lines and different process platforms for the Infrared and Raman spectroscopy. The error of prediction depends strongly on the process platform and is at most 15% in the concentration range higher than 3 g/L (Figure 1).

## Conclusions

The automated, MWP based screening system for suspension cell culture is routinely used in cell culture process development. By extending the fermentation throughput up to 768 reactors in parallel it is necessary to increase the analytical throughput. The application of the Infrared and Raman spectroscopy as HT analytical tools could be successfully shown. The main application shown in this work is the glucose control in the cell culture process. All measurements using Raman spectroscopy were performed fully automated. The Infrared measurements were performed semi-automated dependent on the availability of a suitable system.

The use of the Raman and Infrared spectroscopy enables the high needed analytical throughput of the automated MWP based screening system for suspension cell culture.

## Acknowledgements

The authors would like to thank the Robotic team (J. Hoffmann, G. Pechmann, C. Schuster), all internship and diploma students (R.Wetzel, K. Moeser, P. Linke, S. Spielmann, K. Müller, B. Frommeyer, J.Wisbauer, A.Gutknecht), the Roche Penzberg pilot plant and GMP facility team, all RochePenzberg portfolio project teams and the University of Hannover (Prof. Dr.Thomas Scheper, Dr. D. Solle).
